# Application of the decision tree model in ADHD screening

**DOI:** 10.1192/j.eurpsy.2021.1999

**Published:** 2021-08-13

**Authors:** L.R. Carreiro, M. Silva, M.C. Teixeira

**Affiliations:** Developmental Disorders Graduate Program, Mackenzie Presbyterian University, São Paulo, Brazil

**Keywords:** ADHD, Behavioral profile, Decision tree, Neuropsychological profile

## Abstract

**Introduction:**

Attention Deficit Hyperactivity Disorder (ADHD) is a Neurodevelopmental Disorder characterized by persistent pattern of inattention and hyperactivity / impulsivity. There is considerable difficulty in diagnosing ADHD, mainly to discriminate what could be symptoms arising from ADHD or typical age behaviors. The decision tree model is a statistical algorithm, a predictive model built with comparisons of values for a given objective that can be compared with other constant values, placing these variables in a database at hierarchical levels.

**Objectives:**

This study aims to apply the decision tree model in directing the screening of ADHD complaints to analyze which cognitive and behavioral parameters would be better associations with ADHD accurate diagnosis

**Methods:**

We used a database of research protocol with 202 children assessed with complaints of ADHD and a control group with 185 participants. Decision tree analyzed parameters selected from the cognitive instruments, such voluntary attention, Continuous Performance Test indexes, WCST indexes, Wechsler Intelligence indexes and behavioral scales from CBCL/6-1 and TRF/6-18.

**Results:**

The highlighted results points to WCST index like: “Perseverative answers” and “Perseverative errors” and “learning to learn” joint to “CPT omissions” and behavioral scales as “CBCL ADHD”, and “CBCL Problems of Attention” produces accuracy of diagnosis discrimination from 84.7% to 60% in the precision of the decision tree.

**Conclusions:**

The decision tree and machine learning approaches can be effective in directing the screening of typical ADHD complaints.

**Disclosure:**

No significant relationships.

